# Macrolide Resistance in Adults with Bacteremic Pneumococcal Pneumonia

**DOI:** 10.3201/eid1208.060017

**Published:** 2006-08

**Authors:** Joshua P. Metlay, Neil O. Fishman, Marshall M. Joffe, Michael J. Kallan, Jesse L. Chittams, Paul H. Edelstein

**Affiliations:** * Philadelphia Veterans Affairs Medical Center, Philadelphia, Pennsylvania, USA;; † University of Pennsylvania School of Medicine, Philadelphia, Pennsylvania, USA

**Keywords:** antibacterial agents, microbial drug resistance, Streptococcus pneumoniae, research

## Abstract

We conducted a case-control study of adults with bacteremic pneumococcal pneumonia to identify factors associated with macrolide resistance. Study participants were identified through population-based surveillance in a 5-county region surrounding Philadelphia. Forty-three hospitals contributed 444 patients, who were interviewed by telephone regarding potential risk factors. In multivariable analyses, prior exposure to a macrolide antimicrobial agent (odds ratio [OR] 2.8), prior flu vaccination (OR 2.0), and Hispanic ethnicity (OR 4.1) were independently associated with an increased probability of macrolide resistance, and a history of stroke was independently associated with a decreased probability of macrolide resistance (OR 0.2). Fifty-five percent of patients with macrolide-resistant infections reported no antimicrobial drug exposure in the preceding 6 months. Among patients who reported taking antimicrobial agents in the 6 months preceding infection, failure to complete the course of prescribed drugs was associated with an increased probability of macrolide resistance (OR 3.4).

*Streptococcus pneumoniae* is the leading cause of community-acquired pneumonia in adults. Bacteremic pneumococcal pneumonia is among the most serious forms of pneumococcal disease, and incidence rises steeply with advancing age (*1*). Although considerable controversy exists about the clinical impact of pneumococcal drug resistance (*2*), the prevalence of single-drug and multidrug–resistant pneumococci has increased in the last 2 decades (*3**,**4*). Drug-resistant pneumococci clearly emerge under the selective forces of antibacterial drugs used in the population. Still, the precise nature of these selection mechanisms and the risk associated with different types of exposures are not well defined.

Pneumococcal resistance to macrolides is a problem because macrolides are among the most common oral drugs used to treat patients with community-acquired pneumonia (*5*). A recent study found that patients with macrolide-resistant pneumococcal bacteremia were substantially more likely to have been exposed to macrolide therapy before hospitalization than were patients with macrolide-susceptible pneumococcal bacteremia (*6*). Since most initial therapy of community-acquired pneumonia is empiric, estimating the probability of macrolide-resistant pneumococcal disease is necessary to select appropriate therapy. Indeed, current treatment guidelines recommend not prescribing macrolide therapy alone for patients with community-acquired pneumonia if they report exposure to macrolides within the 3 months preceding the onset of illness (*7*).

We conducted a population-based case-control study to identify clinical and demographic factors independently associated with macrolide-resistant bacteremic pneumococcal pneumonia in adults. We used a detailed multistage interview method to elicit in-depth histories of exposure to antimicrobial agents to examine whether disease probability varied across different patterns of antibacterial drug exposure.

## Methods

### Design

We conducted a case-control study within a network of hospitals conducting prospective population-based surveillance for bacteremic pneumococcal pneumonia in adults in southeastern Pennsylvania from December 1, 2000, to April 17, 2004 (Appendix). Patients were all hospitalized adults with macrolide-resistant bacteremic pneumococcal pneumonia, and controls were all hospitalized adults with macrolide-susceptible bacteremic pneumococcal pneumonia.

### Study Site

This study was conducted within the 5-county region surrounding Philadelphia, Pennsylvania: Bucks, Chester, Delaware, Montgomery, and Philadelphia Counties. The adult population (age >18 years) of this region is 2,881,132 (US Census 2000). At the start of the surveillance period, 46 acute-care hospitals served this region; 43 of them participated in this study. Of the remaining 3 hospitals, 2 were small hospitals closed to external studies, and 1 was a larger academic hospital that was unable to participate.

### Study Participants

Inclusion criteria for the study were persons who 1) were >18 years of age, 2) had at least 1 blood culture that grew *S. pneumoniae* drawn within 48 hours of hospital admission, 3) resided in 1 of the 5 counties, and 4) had a bacterial isolate confirmed in our laboratory as *S. pneumoniae* (see below). Study participants were further restricted on the basis of physician report to those patients with radiographic evidence of an acute respiratory infection. Exclusion criteria for the case-control study included evidence of bacterial meningitis (cerebrospinal fluid [CSF] growth of *S. pneumoniae* or CSF findings compatible with bacterial meningitis) or hospitalization within 10 days preceding the index hospitalization. Study participants who died during hospitalization were included in this study; information about them was collected by interviewing a suitable proxy respondent.

Study participants were identified by microbiology laboratory personnel at each participating hospital. Whenever laboratory personnel identified a blood culture with growth of *S. pneumoniae*, research staff contacted the physician of record to determine the patient's eligibility. Eligible participants (or proxies in cases of mental incompetence or death) were then approached for study enrollment at a time determined by the treating physician (typically after hospital discharge). Participants were mailed informational study materials and then contacted by phone to provide consent for study participation and complete a telephone interview.

### Data Collection

Trained telephone interviewers completed a telephone interview with each study participant that covered demographic and clinical areas. Questions focused on the demographic and clinical status of the patient immediately before hospitalization for pneumococcal pneumonia. A multistep strategy was used to obtain the most complete drug histories possible. A phased approach was employed in which the interviewer first asked open-ended questions about use of drugs, then asked indication-specific questions about medications used (e.g., for respiratory tract infections, urinary tract infection) and, finally, named antimicrobial drugs by brand and generic names while the participant referred to photo hand cards (mailed to the participants in advance) that displayed the study drugs of interest. In prior research, including a study of antimicrobial drug recall, each of these steps has dramatically increased drug recall (*8**–**11*). Participants were asked to distinguish antimicrobial agents that were being taken at the time of hospitalization from those drugs that were taken for illnesses preceding the onset of pneumococcal pneumonia. We focused on patient self-report of prior antimicrobial drug exposure to mimic the information that would be available at the time of diagnosis and empiric treatment decisions. However, we also contacted the primary care physicians of study participants to obtain documentation of antimicrobial agents prescribed in the 6-month period preceding the hospitalization for pneumococcal pneumonia. In addition, since study participants were interviewed at home, we asked them to examine any medication bottles that they still possessed to verify the name of the drug and to determine if any medications were unfinished.

### Microbiologic Data Collection

Pneumococcal blood isolates were transported to a central laboratory at the Hospital of the University of Pennsylvania for analysis. Isolates were re-identified to confirm that they were pneumococci on the basis of colony shape and hemolytic activity, Gram stain appearance, catalase reaction, bile solubility, and optochin susceptibility (*12*).

Confirmed isolates of *S. pneumoniae* were screened for susceptibility to oxacillin, erythromycin, clindamycin, tetracycline, trimethoprim-sulfamethoxazole, and levofloxacin by using the Clinical and Laboratory Standards Institute (CLSI, formerly National Committee for Clinical Laboratory Standards) disk-diffusion procedure (*13*). Isolates that demonstrated reduced susceptibility to any drug were confirmed by using a Food and Drug Administration–cleared and CLSI-compliant microbroth dilution testing method (Sensititer 96 well plate, Trek Diagnostics Systems, Inc., Cleveland, OH, USA) for *S. pneumoniae*. Because the highest erythromycin microbroth MICs that could be measured with the assay were 4 μg/mL, additional testing was performed on all erythromycin-resistant isolates with the Etest method, as recommended by the manufacturer, which includes the use of Mueller-Hinton 5% sheep's blood agar (BBL brand, BD Diagnostic Systems, Sparks, MD, USA) and incubation in 5% CO_2_ for 20 to 24 h. Carbon dioxide incubation, required for the optimal growth of many pneumococci on solid media, increases erythromycin MICs by ≈1 doubling dilution and therefore changes the erythromycin MIC breakpoints to <0.5, susceptible, 1 intermediate, and >2 resistant (*14*). For the purposes of this study, we combined isolates with intermediate susceptibility and resistance to erythromycin as erythromycin-resistant cases for the case-control study. However, only 1 isolate among these cases had an erythromycin MIC = 1.0 μg/mL; the remainder had MICs >1.0 μg/mL.

All pneumococcal isolates were serotyped according to standard methods by using the Quellung reaction (*15**–**17*). All sera were purchased from the Statens Serum Institut (WHO Collaborating Centre for Reference and Research on Pneumococci) and included 14 pooled sera, 62 factor sera, and 22 type sera.

### Data Analysis

We calculated descriptive statistics for case-patients and controls and compared the distribution of demographic and clinical characteristics by using χ^2^ test statistics. We compared the self-reported patterns of prior antimicrobial drug exposure between case-patients and controls, distinguishing antimicrobial agents that were taken before the onset of the illness and those taken during the current illness up to the time of hospitalization.

Multivariable analyses were completed with logistic regression. We included as candidate risk factors all variables that were significantly associated with case versus control status at p<0.10 in bivariate analyses. We developed a final model using backward elimination, with variables with p>0.05 eliminated from the model. Associations between risk factors and macrolide-resistant bacteremic pneumococcal pneumonia that remained in the model are presented as odds ratios (ORs) and 95% confidence intervals (CIs). A separate model examining patterns of antimicrobial drug exposure was constructed restricted to those participants who reported >1 prior exposure to antimicrobial drugs during the 6 months preceding onset of illness. This study was approved by the institutional review boards at the University of Pennsylvania School of Medicine and each participating hospital.

## Results

From December 1, 2000, through April 17, 2004, a total of 1,209 cases of pneumococcal bacteremia among adults in the 5-county region were identified. Excluding patients without a concurrent diagnosis of pneumonia, with a concurrent diagnosis of meningitis, residence outside the 5-county region, or hospitalization within 10 days of the episode yielded 956 eligible participants. We enrolled 444 (46%). Reasons for nonenrollment included physician refusal (26%), patient or proxy refusal (36%), and inability to locate the patient or family (24%).

Seventy- six patients (17%) had erythromycin-resistant infections (MIC_50_ = 8.0 μg/mL, MIC_90_ = 256 μg/mL, range 1–256 μg/mL) and were selected as the case-patients for this study (Figure). As expected, 22 of 23 isolates with erythromycin MICs >64 μg/mL were also clindamycin resistant (MLS_B_ phenotype), and 49 of 53 isolates with erythromycin MICs <32 μg/mL were clindamycin susceptible and comprised the M phenotype (*18*). Compared to the pneumococcal isolates from patients with erythromycin-susceptible infections, isolates from patients with erythromycin-resistant infections were more likely to have reduced susceptibility to penicillin (75% vs. 11%), tetracycline (38% vs. 1%), and trimethoprim-sulfamethoxazole (62% vs. 10%) (all p<0.0001). However, susceptibility to fluoroquinolones (specifically levofloxacin) was the same for erythromycin-resistant and erythromycin-susceptible isolates (1% of erythromycin-susceptible and -resistant isolates were resistant to levofloxacin, p = 0.82). Compared to the erythromycin-susceptible isolates, erythromycin-resistant isolates were more than twice as likely to belong to 1 of the 7 serotypes contained within the new pneumococcal conjugate vaccine (45% vs. 22%, p<0.0001).

**Figure:**
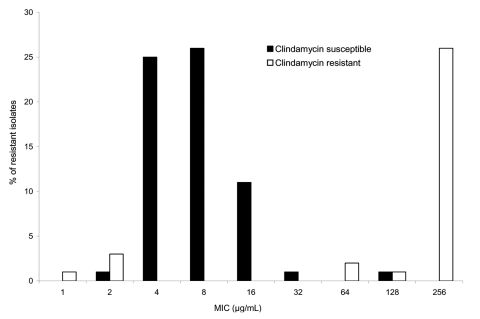
MIC distribution of resistant isolates. This figure displays the proportion of resistant isolates at each MIC. Isolates with clindamycin susceptibility are analyzed separately from isolates with clindamycin resistance. The total number of isolates is 76.

### Demographic and Clinical Risk Factors

Among potential demographic factors, only race and ethnicity were significantly associated with erythromycin resistance. White patients were more likely to have a resistant infection compared to patients self-reporting other racial categories (Asian, African American or Black, and Native Hawaiian or other Pacific Islander) (OR 1.8, 95% CI 1.0–3.1), and Hispanic patients were more likely to have a resistant infection compared to non-Hispanic patients (OR 3.1, 95% CI 1.1–8.8). Patient age, sex, education, income, and urban versus suburban residence were not significantly associated with the probability of an erythromycin-resistant infection (Table 1).

**Table 1:** Demographic and clinical characteristics of cases and controls*

Characteristic		Macrolide resistant, n = 76 (%)	Macrolide susceptible, n = 368 (%)	OR	95% CI	p value
Demographic factors						
	Age >65 y	40 (53)	163 (44)	1.4	0.9–2.3	0.19
	Male sex	36 (48)	169 (46)	1.1	0.7–1.8	0.73
	White race	57 (76)	236 (64)	1.8	1.0–3.1	0.048
	Hispanic ethnicity	6 (8)	10 (2)	3.1	1.1–8.8	0.026
	Nursing home residence	4 (5)	23 (6)	0.8	0.2–2.6	0.71
	Annual income >US $25,000	30 (55)	136 (49)	1.2	0.7–2.2	0.46
	More than high-school education	53 (72)	236 (64)	1.4	0.8–2.4	0.24
	Children (< 6 y) in home	12 (16)	62 (17)	0.9	0.4–1.9	0.82
	Philadelphia residence	29 (39)	176 (48)	0.7	0.4–1.2	0.15
Clinical factors						
	HIV infection	5 (7)	45 (12)	0.5	0.2–1.3	0.17
	Active smoking	25 (33)	107 (29)	1.2	0.7–2.1	0.45
	Asthma	19 (25)	73 (17)	1.4	0.8–2.5	0.28
	Chronic bronchitis/emphysema	23 (31)	68 (18)	2.0	1.1–3.4	0.017
	Coronary artery disease	9 (12)	67 (18)	0.6	0.3–1.3	0.20
	Congestive heart failure	8 (11)	72 (20)	0.5	0.2–1.1	0.069
	History of stroke	2 (3)	43 (12)	0.2	0.1–0.9	0.019
	Diabetes mellitus	12 (17)	89 (24)	0.7	0.4–1.3	0.20
	Chronic renal disease	3 (4)	40 (11)	0.3	0.1–1.1	0.068
	Active cancer	15 (20)	51 (14)	1.6	0.8–3.0	0.17
	Chronic liver disease	7 (9)	43 (12)	0.8	0.3–1.8	0.56
	Prior influenza vaccination†	46 (61)	178 (48)	1.7	1.0–2.8	0.039
	Prior pneumococcal vaccination‡	35 (47)	198 (54)	0.8	0.5–1.2	0.27

*OR, odds radio; CI, confidence interval. p value based on χ^2^ test. †In the 12 months preceding the date of infection. ‡At any time before the date of infection.

Among potential clinical factors, a history of chronic bronchitis or emphysema was associated with an increased probability of erythromycin resistance (OR 2.0, 95% CI 1.1–3.4), and a history of stroke was associated with a reduced probability of resistance (OR 0.2, 95% CI 0.1–0.9). Patient report of receiving influenza vaccination in the prior year was associated with an increased probability of resistance (OR 1.7, 95% CI 1.0–2.8). Other coexisting conditions, including HIV infection, asthma, and diabetes mellitus, were not significantly associated with susceptibility of the pneumococcal isolate (Table 1).

Patient report of any exposure to antimicrobial agents during the 6 months preceding the episode of bacteremic pneumococcal pneumonia was associated with a >2-fold increase in the odds of having an erythromycin-resistant isolate (OR 2.2, 95% CI 1.3–3.7). Fifty-five percent of patients with erythromycin-resistant infections did not report any antimicrobial drug exposures in the preceding 6 months. Among different classes of antimicrobial agents, prior exposure to macrolides and quinolones was each associated with an increased probability of macrolide resistance, but reported exposure to penicillins, cephalosporins, and tetracyclines was not associated with an increased probability (Table 2). The major macrolide drugs reported by patients were azithromycin (58%) and clarithromycin (30%). The major fluoroquinolone drugs reported by patients were levofloxacin (49%) and ciprofloxacin (40%). Physicians provided outpatient medical record information on antimicrobial drug exposure for 342 (77%) of the 444 study participants. Based on physician report, documented prescription of an antimicrobial agent in the 6 months preceding the episode of bacteremic pneumococcal pneumonia was associated with an almost 2-fold increase in the odds of having an erythromycin-resistant isolate, but this finding did not achieve significance (OR 1.7 95% CI 1.0–3.1).

**Table 2:** Patterns of prior antimicrobial drug exposure for patients and controls*

Antimicrobial agent exposure	Macrolide resistant, n = 76 (%)	Macrolide susceptible, n = 368 (%)	OR	95% CI	p value
Any in prior 6 mo†	34 (45)	101 (27)	2.2	1.3–3.7	0.002
Any macrolide in prior 6 mo	14 (19)	29 (8)	2.7	1.3–5.4	0.004
Any quinolone in prior 6 mo	14 (19)	33 (9)	2.3	1.2–4.6	0.013
Any penicillin in prior 6 mo	8 (11)	36 (10)	1.1	0.5–2.5	0.81
Any cephalosporin in prior 6 mo	4 (5)	15 (4)	1.3	0.4–4.1	0.62
Any tetracycline in prior 6 mo	1 (1)	3 (1)	1.6	0.2–16.1	0.66
No. antimicrobial agents in 6 mo					
None	41 (55)	261 (71)	Referent		
1 prescription	18 (24)	75 (20)	1.5	0.8–2.7	0.20
>2 prescriptions	16 (21)	33 (9)	3.0	1.5–6.0	0.002
Did not complete last prescription	9 (12)	14 (4)	3.5	1.4–8.3	0.004
On antimicrobial agent at admission	4 (5)	7 (2)	2.9	0.8–10.2	0.083
Time since antimicrobial agent‡					
No prior drug use	44 (59)	277 (75)	Referent		
<3 mo	22 (29)	62 (17)	2.2	1.2–4.2	0.006
4–6 mo	9 (12)	30 (8)	1.7	0.8–4.5	0.12

*Odds ratio (OR) and 95% binomial confidence interval (95% CI). P value based on χ^2^ test. †Does not include antimicrobial agents that patient was taking at time of admission for bacteremic pneumococcal pneumonia. ‡For patients on >1 antimicrobial agent in previous 6 months, this represents time since most recent course of drugs.

Among patients who reported any prior exposure to antimicrobial drugs, the probability of macrolide resistance increased with patient report of increasing number of prior courses of drugs. Patients who reported only 1 prior course had a 1.5-fold increased odds of a resistant infection, whereas patients who reported >2 courses of antimicrobial agents had a 3.0-fold increased odds of a resistant infection. In addition, the relationship of antimicrobial drug exposure to the probability of an erythromycin-resistant infection was time sensitive: patients with such exposure within 3 months of infection had significantly increased odds of resistant infection, whereas patients exposed during the 4–6 months preceding infection did not have significantly increased odds of resistant infection. Finally, among patients who had at least 1 course of drugs, reporting that they did not finish the prescribed course was associated with >3-fold increased odds of a resistant infection compared to that for patients who reported completing the most recent course of antimicrobial agents (OR 3.5, 95% CI 1.4–8.3).

In multivariable analysis, prior exposure to macrolides (OR 2.8), prior influenza vaccination (OR 2.0), and Hispanic ethnicity (OR 4.1) were independently associated with an increased probability of macrolide resistance; a history of stroke (OR 0.2) was independently associated with a reduced probability of macrolide resistance (Table 3). All patients with a macrolide-resistant infection reported >1 of these 4 factors (prior exposure to macrolides, prior flu shot, Hispanic ethnicity, or no history of stroke). However, 97% of all study patients reported >1 of these 4 factors (data not shown). Among patients who reported at least 1 course of an antimicrobial agent in the 6 months preceding infection, the only significant characteristic of prior exposure was the patient's report that he or she failed to complete the full prescription (OR 3.4, 95% CI 1.2–9.9).

**Table 3:** Independent risk factors for macrolide-resistant bacteremic pneumococcal pneumonia*

Risk factor	OR (95% CI)	p value
Macrolide <6 mo before infection	2.8 (1.4–5.8)	0.005
Influenza vaccination <1 mo before infection	2.0 (1.2–3.3)	0.013
Hispanic ethnicity	4.1 (1.4–12.5)	0.011
Prior stroke	0.2 (0.04–0.8)	0.021

*Odds ratio (OR), 95% confidence intervals (CI), and p value from logistic regression with all listed factors in the model.

## Discussion

In this case-control study of 444 adults with bacteremic pneumococcal pneumonia, we found that exposure to macrolides in the 6 months preceding infection, a history of influenza vaccination in the 12 months preceding infection, and Hispanic ethnicity were all independently associated with an increased probability of an erythromycin-resistant infection. However, most patients with erythromycin-resistant pneumococcal infections did not report any antimicrobial drug exposures in the 6 months preceding infection.

That prior antibacterial drug exposure is a risk factor for drug-resistant pneumococcal infections is supported by mathematical models and most empiric studies. Numerous studies have suggested a relatively strong association between prior antimicrobial drug use and the subsequent development of invasive infections due to penicillin-resistant pneumococcal infections (*19**–**24*). A recent case-control study comparing penicillin-susceptible to penicillin-nonsusceptible isolates from patients with pneumococcal bacteremia identified prior exposure to β-lactams, sulfonamides, and macrolides as risk factors; fluoroquinolone exposure was not a risk factor. These risk factors remained relevant up to 6 months before infection (*25*). Similarly, in another recent study of invasive pneumococcal disease comparing patients with macrolide-resistant isolates to macrolide-susceptible isolates, exposure to each of the following drugs was associated with an increased probability of a macrolide-resistant infection: penicillin, trimethoprim-sulfamethoxazole, clarithromycin, or azithromycin (*26*). While our current study found that exposure to the macrolide drug class had the strongest association with the odds of a macrolide-resistant infection, the sample was too small to separately analyze the risk associated with different drugs within that class. However, given that antimicrobial drug exposure is common, research on modifiable risk factors for drug-resistant pneumococcal infections needs to focus on different patterns of exposure, both in terms of specific drugs selected and the dose and duration of administration. Among patients with a prior exposure to antimicrobial agents, reporting that they did not complete the course was significantly associated with the odds of a macrolide-resistant infection. Future studies correlating duration of therapy with risk for colonization with macrolide-resistant pneumococci would be useful to further explore this phenomenon.

Additional risk factors associated with drug-resistant pneumococcal infections have been reported to include extremes of age, attendance in daycare, having a household member in daycare, and coexisting illnesses, particularly HIV infection (*4**,**21**,**27**–**29*). However, many of these risk factors may be identified only because they are associated with higher probabilities of prior antimicrobial drug exposure, which may have been incompletely measured by our questions on prior drug use. In this study, for example, patients who report prior influenza vaccination may have increased access to health providers or increased frequency of respiratory infections, both factors that would likely increase the probability of prior antimicrobial drug exposure. Similarly, while we asked many questions about prior antimicrobial drug exposure, the observed association between erythromycin resistance and Hispanic ethnicity may be confounded by increased access to antimicrobial drugs through nontraditional sources (such as markets), where they may be less readily identified as antimicrobial agents (*30*). On the other hand, the identification of antimicrobial drug–independent risk factors would suggest that an additional mechanism, specifically increased exposure to persons with antimicrobial drug–resistant bacteria, is a factor promoting the emergence of macrolide-resistant pneumococcal infections. In this regard, the reduced probability of resistant infections seen in patients with a history of stroke might relate to relative social isolation in this population, which would reduce exposure to persons carrying drug-resistant pneumococci. Finally, some of the observed associations may be due to random (type I) error and represent false-positive results.

We did not enroll all patients with bacteremic pneumococcal pneumonia. Therefore, selection bias may have affected our assessment of different risk factors, particularly if enrollment differed for participants with macrolide-resistant and –macrolide-susceptible infections. Our analysis of the drug susceptibility of isolates from nonenrolled patients showed that the proportion of erythromycin-resistant isolates was not significantly different between enrolled and nonenrolled patients (data not shown). In addition, as pointed out by others, the selection of control groups affects the interpretation of results (*31*). In this study, we used patients with antimicrobial drug–susceptible pneumococcal infections as the control group to identify factors that might distinguish patients with pneumococcal infections at the time of treatment decisions. Finally, we relied primarily on patient self-report to identify prior antimicrobial drug exposures and the patterns of these exposures. We used a multistage, previously validated approach to measure exposure. Moreover, patient report is typically the source of information for providers at the time of treatment decisions. Although measurement error may have introduced bias in our risk estimates, the level of association between prior antimicrobial drug exposure and the odds of a macrolide-resistant infection were quantitatively similar when we used information from outpatient medical records.

More broadly, this study demonstrates that among patients with pneumococcal disease, patients with self-reported prior exposure to antimicrobial drugs, particularly macrolides, have an increased probability of infection with macrolide-resistant pneumococci. In addition, additional courses of antimicrobial drugs increase the probability of a drug-resistant infection. However, most patients with macrolide-resistant infections did not report any prior antimicrobial drug exposures. As a result, empiric therapy should be predominately guided by local susceptibility data rather than specific host characteristics.

## Appendix

The Delaware Valley Case Control Network includes the following physicians and laboratory directors listed with their respective hospitals.

Robert Dee, Herbert Auerbach (Abington Memorial Hospital); Jerry Zuckerman, Ierachmiel Daskal (Albert Einstein Medical Center); John Bartels, Stephen B. Chasko (Brandywine Hospital); Albert Keshgegian, Olarae Giger (Main Line Clinical Laboratory); Peter Spitzer, Bryn Mawr Hospital); Lawrence Livornese, (Lankenau Hospital); David Trevino (Paoli Hospital); Abby Huang, David Wright (Central Montgomery Medical Center); Dorothy Slavin, Mark Ingerman, Jerome Santoro, Lawrence Livornese, Ru Lin Ko Tung (Chestnut Hill Hospital); John Roberts, Jim Heald (Chester County Hospital); William Ravreby, Harvey B. Spector (Crozer Chester Medical Center, Taylor Hospital); Margaret Hessen (Springfield Hospital); Lawrence M. Matthews, Margaret Hessen (Delaware County Memorial Hospital); David Loughran, Rose M. Kenny (Doylestown Hospital); Donald Marcus, Xiaoli Chen (Elkins Park Hospital); Richard Tepper, Ila Mirchandani (Jeanes Hospital); Peter Axelrod (Fox Chase Cancer Center); Donald Marcus, Howard Elefant (Frankford Hospital Torresdale Division, Frankford Hospital Bucks County Campus, Frankford Hospital Frankford Division); Bonnie Rabinowitch, Fernando U. Garcia (Graduate Hospital); Abby Huang, Irwin Hollander (Grand View Hospital); Young S. Kim, Christopher Emery (Hahnemann University Hospital); Robert Dee, Pantaleon Fagel (Holy Redeemer Hospital and Medical Center); Paul Edelstein (Hospital of the University of Pennsylvania and Presbyterian Hospital); Paul McGovern (Presbyterian Hospital); Lorenzo M. Galindo (Mercy Fitzgerald, Mercy Suburban, Mercy Hospital of Philadelphia); William McNamee (Mercy Fitzgerald, Mercy Hospital of Philadelphia); Wayne Miller (Mercy Suburban Hospital); Robert Measley, Harvey J. Bellin (Methodist Hospital); David S. Fox, Paul Belser (Montgomery Hospital); Michael Braffman, John Stern, Gary Stopyra (Pennsylvania Hospital); Raymond Kovalski, Leonas Bekeris (Phoenixville Hospital); Raymond Kovalski, Dante DiMarzio (Pottstown Memorial Hospital); William McNamee, Susan Yaron (Riddle Memorial Hospital); Lawrence Livornese, Pradeep Bhagat (Roxborough Memorial Hospital); John Bartels, James Monihan (Jennersville Regional Medical Center); Robert Measley, John McCormick (St Agnes Medical Center); Abby Huang , David Steinberg, (St Luke's Quakertown); Donald Marcus, Zenon Gibas, Helen Kroh (St Mary Medical Center); Peter Axelrod, Allan Truant, Jamshid Moghaddas (Temple University Hospital, Northeastern Hospital, Episcopal Hospital); Jerry Zuckerman (Northeastern Hospital); Gregory Kane, Fred Gorestein, Donald Jungkind (Thomas Jefferson University Hospital); Donald Stieritz (Philadelphia VA Medical Center); David Loughran, Manjula Balasubramanian (Warminster Hospital).
